# Correlative multi-scale cryo-imaging unveils SARS-CoV-2 assembly and egress

**DOI:** 10.1038/s41467-021-24887-y

**Published:** 2021-07-30

**Authors:** Luiza Mendonça, Andrew Howe, James B. Gilchrist, Yuewen Sheng, Dapeng Sun, Michael L. Knight, Laura C. Zanetti-Domingues, Benji Bateman, Anna-Sophia Krebs, Long Chen, Julika Radecke, Vivian D. Li, Tao Ni, Ilias Kounatidis, Mohamed A. Koronfel, Marta Szynkiewicz, Maria Harkiolaki, Marisa L. Martin-Fernandez, William James, Peijun Zhang

**Affiliations:** 1 Division of Structural Biology, Wellcome Trust Centre for Human Genetics, University of Oxford, Oxford, UK; 2 Diamond Light Source, Harwell Science and Innovation Campus, Didcot, UK; 3 Department of Structural Biology, University of Pittsburgh, Pittsburgh, Pennsylvania, USA; 4 Sir William Dunn School of Pathology, University of Oxford, Oxford, UK; 5 Central Laser Facility, Science and Technology Facilities Council, Rutherford Appleton Laboratory, Didcot, Oxfordshire UK; 6 Murray Edwards College, University of Cambridge, Cambridge, UK

**Keywords:** Pathogens, SARS-CoV-2, Virus structures, Cryoelectron tomography

## Abstract

Since the outbreak of the SARS-CoV-2 pandemic, there have been intense structural studies on purified viral components and inactivated viruses. However, structural and ultrastructural evidence on how the SARS-CoV-2 infection progresses in the native cellular context is scarce, and there is a lack of comprehensive knowledge on the SARS-CoV-2 replicative cycle. To correlate cytopathic events induced by SARS-CoV-2 with virus replication processes in frozen-hydrated cells, we established a unique multi-modal, multi-scale cryo-correlative platform to image SARS-CoV-2 infection in Vero cells. This platform combines serial cryoFIB/SEM volume imaging and soft X-ray cryo-tomography with cell lamellae-based cryo-electron tomography (cryoET) and subtomogram averaging. Here we report critical SARS-CoV-2 structural events – e.g. viral RNA transport portals, virus assembly intermediates, virus egress pathway, and native virus spike structures, in the context of whole-cell volumes revealing drastic cytppathic changes. This integrated approach allows a holistic view of SARS-CoV-2 infection, from the whole cell to individual molecules.

## Abstract (web summary)

In this study, Peijun Zhang and colleagues use cryoFIB/SEM volume imaging and soft x-ray cryo-tomography with cryo-electron tomography (cryoET) of cellular periphery, lamellae, and subtomogram averaging to place critical structural events in the SARS-CoV-2 infection cycle in the context of whole-cell images.

## Introduction

Since December 2019, the world has been in the middle of what has been dubbed the “greatest pandemic of the century.” The etiological agent was severe acute respiratory syndrome coronavirus 2 (SARS-CoV-2) and the disease caused by it is coronavirus disease 2019^1–3^. Coronaviruses are small enveloped viruses with positive non-segmented RNA genome. Among RNA viruses, coronaviruses bear one of the largest genomes and their replication is complex involving frameshift slipping and replicase jumps with abundant RNA duplexes being generated. Coronaviruses, like most RNA viruses, induce the development of a range of membrane compartments that seclude and protect viral components contributing to increased replication efficiency and innate immune recognition escape^4–10^.

All coronavirus structural proteins arise from the translation of positive-sense subgenomic RNA fragments, generated from minus-sense subgenomic RNAs fragments, which are in turn generated by replicase jumps when the viral genome is transcribed. Of these, the S protein makes the viral spike, responsible for cellular attachment, entry, and fusion. It adopts two main conformations: prefusion, composed of trimers of subunits S1 and S2, and postfusion, a non-active conformation composed solely of S2^11–18^. The N protein is responsible for encapsidating and protecting the genomic viral RNA (vRNA), forming ribonucleoprotein complexes that reside in the internal space of the viral particle. The E protein is the smallest of the structural proteins and is thought to act as an ion channel^19^. Finally, the M protein is the most abundant in SARS-CoV-2 and is a transmembrane protein that lines the internal surface of the virus lipid membrane^20^.

The SARS-CoV-2 cycle starts when S interacts with ACE2 on the host cell surface^13–15,17,21^. This interaction can either be followed by S2’ cleavage at the cell surface by the host cell protease TMPRSS2 or trigger the endocytosis of the viral particle, when TMPRSS2 is not present^13^. Upon a second still not completely characterized trigger, which may be the S2’ site cleavage and/or endosomal acidification, the spike changes conformation and inserts its fusogenic peptide into the host membrane to fuse it with the viral envelope, after which the spike adopts the postfusion conformation^11,12,22,23^. The viral contents are then released into the cytoplasm, and the precursor polyproteins Pp1a and Pp1ab are synthetized. Non-structural proteins 3, 4, and 6 (nsp3, nsp4, and nsp6), which are part of the replicase polyproteins Pp1a/Pp1ab, induce the formation of secluded, often interconnected, membranous compartments known as double membrane vesicles (DMVs)^24–26^. The DMVs compartmentalize SARS-CoV-2 replication transcription complexes and are the main cellular compartments where viral genome replication and transcription takes place^4,7^. These compartments were thought to be sealed, raising the mystery of how the mRNAs could reach the cytoplasm to be translated by the cellular ribosomes. Recently, however, a molecular pore has been described in murine hepatitis coronavirus (MHV) and SARS-CoV-2 that can serve as export portal for the mRNA and positive-strand viral genome copies^27^. The final assembly of the viral particle is thought to take place at modified cellular membranes derived from the endoplasmic reticulum (ER), Golgi, and ER–Golgi intermediate compartment (ERGIC) and viral release through exocytosis based on studies of other coronaviruses^4,28–31^. A recent fluorescence microscopic study also suggested SARS-CoV-2 release through lysosomal exocytosis^32^. Although there have been intense structural studies on recombinant viral components and purified inactivated viruses^13–15,17,21,33–35^, structural investigation of the SARS-CoV-2 replication process in the native cellular context is scarce^4,27^, and viral assembly and egress are still not well understood.

In this study, we exploited a unique correlative multi-modal multi-scale cryo-imaging approach to investigate SARS-CoV-2 replication in Vero cells under near-native conditions. This approach empowers a holistic view of SARS-CoV-2 infection, from the whole cell to individual virus spike molecules, revealing pathways of SARS-CoV-2 assembly and egress and cytopathic effects of SARS-CoV-2 infection. These results substantiate previous findings obtained by thin-sectioning electron microscopy (EM) and serial focused ion beam scanning electron microscopy (FIB/SEM) of stained plastic-embedded samples^36^ and further expand our knowledge of SARS-CoV-2 assembly and egress^4^, as well as its presence in other membrane compartments. It validates serial cryoFIB/SEM and soft X-ray tomography as techniques to investigate whole-cell morphology correlated with high-resolution cryo-electron tomography (cryoET), with the advantages of frozen-hydrated conditions and fast and straightforward sample preparation.

## Results

### SARS-CoV-2 replication induces profound cytopathic effects in host cells

To image and investigate SARS-CoV-2 replication in the context of the intracellular universe under near-physiological conditions, we infected Vero cells grown on indexed EM grids with SARS-CoV-2 at 0.1 and 0.5 multiplicity of infection (MOI). At 24 h post infection (hpi), the cells were fixed with 4% paraformaldehyde and plunge frozen in liquid ethane. As illustrated in the workflow (Supplementary Fig. 1), EM grids containing SARS-CoV-2-infected cells were first imaged in a Titan Krios to identify infected cells (39.2% for MOI of 0.1 and 45.4% for MOI 0.5) through the detection of abundant viral particles in the cell periphery. SARS-CoV-2 replication kinetics in Vero cells is shorter than 24 h^30^. Therefore, our sample likely contains a mix of infected cells at different replication cycle stages. CryoET tilt series were collected at the periphery of infected cells. The grids were then transferred to a FIB/SEM dual-beam instrument and the same infected cells were imaged with serial cryoFIB/SEM volume imaging^37^ or cryoFIB milling of cellular lamellae at the target region, where additional cryoET tilt series were collected^38^. In parallel, we imaged additional infected cells by soft X-ray cryo-tomography^39^. The quantification of infected and uninfected cells and associated datasets, as well as quantitative analyses for the different observations, is summarized in Supplementary Table 1. These imaging modalities provide the necessary structural and ultrastructural information at different length scales to visualize the infecting viruses in their cellular context and are highly complementary. Indeed, such a unique approach enabled the direct visualization of the SARS-CoV-2 replication and cytopathic effects in a multi-modal, multi-scale, and correlative manner.

**Fig. 1:**
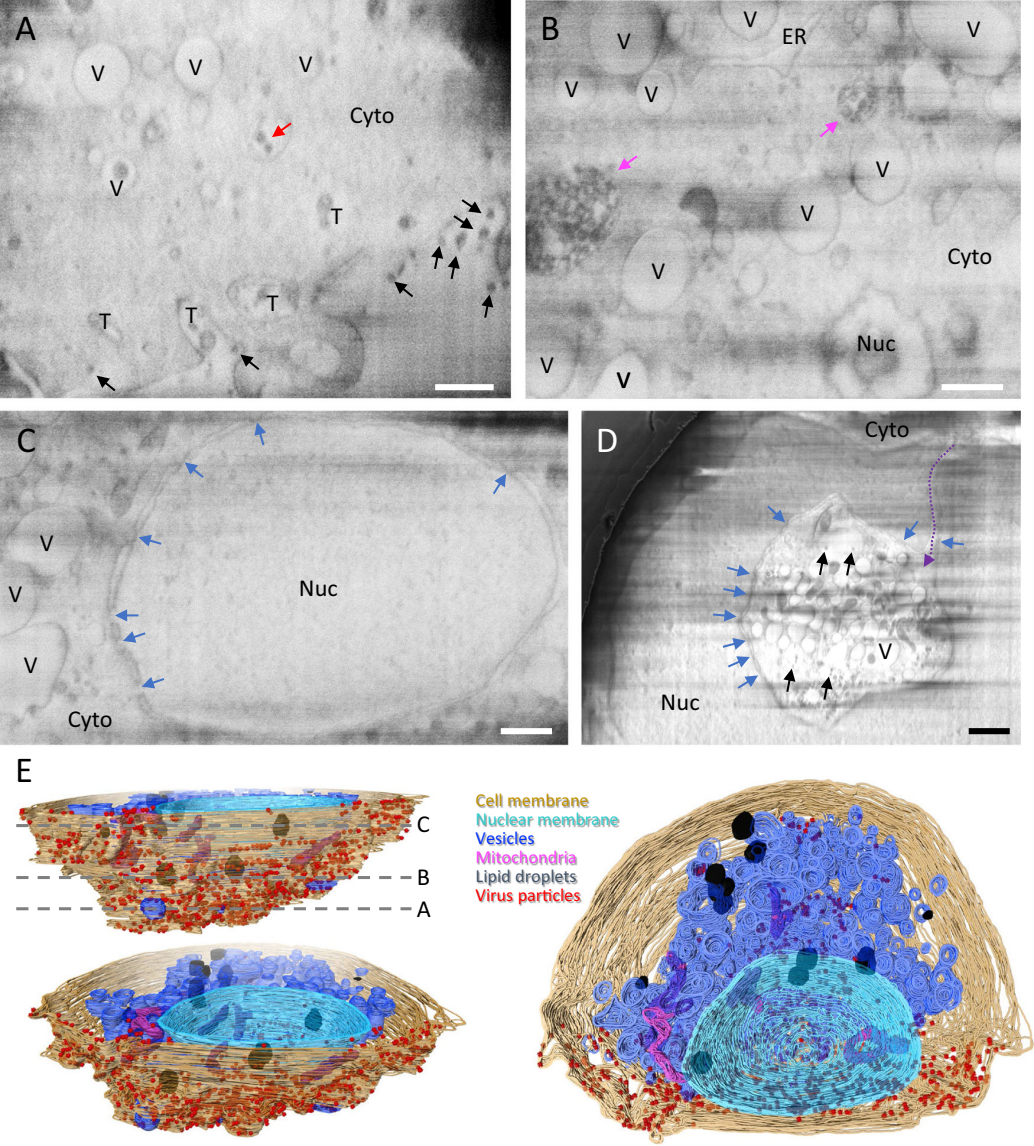
Serial cryoFIB/SEM volume imaging of the entire SARS-CoV-2-infected cell. **A**–**D** Representative cryoFIB/SEM slices of a SARS-CoV-2-infected cell at the cell periphery (**A**), cytoplasm (**B**), cell nucleus (**C**), and invagination of cytoplasm into the nuclear space (note, from a different cell) (**D**). Scale bars, 500 nm in **A**–**C**, 1 µm in **D**. Black and red arrows, extracellular and intracellular virus particles, respectively; blue arrows, nuclear pores; pink arrows, complex membrane compartment; dashed purple arrow, invagination path. V vesicles, T tunnels, Nuc nucleus, Cyto cytoplasm, ER endoplasmic reticulum. **E** Surface rendering of the segmented volume of SARS-CoV-2-infected cell shown in **A**–**C**. Segmented organelles and virus particles are labeled with the colors indicated. The dashed lines (**E**, top left panel) indicate the positions of slices shown in **A**–**C**, respectively.

Compared to uninfected cells (Supplementary Fig. 2 and Supplementary Movie 1), serial cryoFIB/SEM whole-cell imaging of five SARS-CoV-2-infected cells display an extensive array of cytopathological alterations, as illustrated in Fig. 1 and Supplementary Movies 2–6. At the cell periphery, there were numerous electron-dense particles with size consistent to SARS-CoV-2 viruses (Fig. 1A, black arrows). Many membrane tunnel-like structures were observed extending deep into the cell from the cell membrane toward the cytoplasm, containing virus-like particles (Fig. 1A, “T”, and Supplementary Movie 2). These tunnel-like structures resemble those observed in HIV-1-infected macrophages, through which viruses exit the infected cell^40^. In addition, electron-dense virus-like particles were also found within intracellular vesicles that are not connected to cell membrane (Fig. 1A, red arrow). Deeper into the cell, we found that much of the cytoplasm (Fig. 1B), especially the perinuclear region (Fig. 1C), is occupied with numerous vesicles “V” (Supplementary Movies 2–5). Nuclear pores are clearly distinguishable in both SARS-CoV-2-infected and uninfected cells (Fig. 1C and Supplementary Fig. 2A, C, blue arrows). We observed electron-dense complex membranous compartments in infected cells (Fig. 1B, pink arrows). Similar structures has been previously observed and described as large viral-containing vesicles (LVCVs) in thin sections of stained plastic-embedded cells^32^. A more striking feature observed in infected cells is the cytopathic damage to the nucleus compared to the control cells, where, in extreme cases, nearly a half of the nucleus has been taken up by the invaginated cytoplasm (Fig. 1D, Supplementary Movie 6, and Supplementary Fig. 3G). Such cytoplasm invagination was also noticed in EM of stained plastic sections of SARS-CoV-2-infected lung cells and enterocytes^36,41^, suggesting a strong cytopathic effect from SARS-CoV-2 infection.

Exploiting high-throughput whole-cell imaging capability offered by soft X-ray cryo-tomography, we analyzed 5 infected cells that had been identified and confirmed of SARS-CoV-2 infection by cryoET imaging at the cell periphery, in addition to 12 uninfected cells from control grids. At the whole-cell level, soft X-ray images show substantial changes in mitochondria, as long tubular-shaped mitochondria present throughout the cytoplasm in the uninfected cells (Supplementary Fig. 3A, C, yellow arrows) were rarely seen in infected cells (Supplementary Fig. 3B, D). We observed a substantial decrease in the number of mitochondria in SARS-CoV-2-infected cells (15.4 ± 10.2 per tomogram in SARS-CoV-2 infected vs 91.3 ± 32 in control cells, *p* = 0.0001; Supplementary Fig. 4A). This is in agreement with previous findings describing alterations of the mitochondrial network caused by SARS-CoV-2 infection of lung cells^36^. Measurements from manually segmented whole-cell cryoFIB/SEM volumes of three infected cell and two uninfected cell showed that the length of mitochondria in SARS-CoV-2-infected cells is significantly shorter than those in control cells (0.60 ± 0.70 μm in SARS-CoV-2 infected vs 0.90 ± 1.14 μm in control cells, *p* = 0.0012; Supplementary Fig. 4B), without a significant reduction of the mitochondria volume (0.058 ± 0.196 μm^3^ in SARS-CoV-2 infected vs 0.141 ± 0.763 μm^3^ in control cells, *p* = 0.113; Supplementary Fig. 4C), suggesting a change in mitochondria morphology. We also observed numerous vesicles at perinuclear regions (Supplementary Fig. 3F) whose size is consistent with DMVs and cytoplasmic invaginations (Supplementary Fig. 3G) in the infected cells. Altogether, these cryo-imaging results substantiate and extend previous findings of cytopathological alterations associated with SARS-CoV-2 infection^30,36,41^.

### SARS-CoV-2 RNA synthesis and transport

**Fig. 2:**
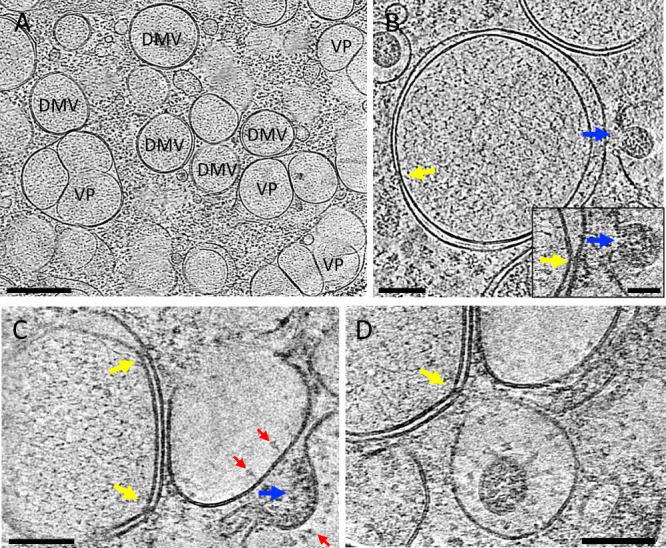
SARS-CoV-2 genome replication and RNA synthesis. **A** Cryo-tomogram slice of cell lamella depicting double membrane vesicles (DMVs) and vesicle packets (VPs). **B** Cryo-tomogram slice of a DMV at high magnification. Inset depicts detail of a DMV portal next to a virus assembly site, viewed at a different angle. **C**, **D** Portals on DMVs next to assembly sites. DMV double membrane vesicle, VP vesicle packet. Yellow arrows, DMV portals; blue arrows, viral assembly sites; red arrows, viral spikes. Scale bars are 500 nm in **A**, 100 nm in **B**, 50 nm in **B** inset, 100 nm in **C**, **D**.

The first step in SARS-CoV-2 infection is viral genome replication. Coronaviruses have evolved a sophisticated RNA replication strategy for the generation of the genomic and subgenomic RNAs relying heavily on double-stranded RNA intermediaries, which are potent activators of RIG-I and MDA-5^42–44^. Thus, cellular compartmentalization of vRNA may serve as an innate immune evasion strategy. DMVs are induced during the replication of a variety of positive RNA viruses^5,6,8–10^ and were identified as the main compartment where vRNA synthesis occurs for coronaviruses^4,7^. Indeed, cryoET of cell lamella revealed that abundant intracellular vesicles observed in the three-dimensional (3D) volume of infected cells (Fig. 1B and Supplementary Movies 2–5) are DMVs likely containing vRNA transcripts as previously suggested^4,7,9,45^ (Fig. 2A–C and Supplementary Movie 7). There are also a substantial amount of vesicle packets (VPs; Fig. 2A). Since the sample was cryofixed 24 hpi, this is consistent with a previous observation that the number of VPs increases with the time of infection^7^. The presence of abundant viral particles in the exterior together with the presence of VP suggest that these cells are in the late stages of viral replication; nonetheless, they could also be in a second replication cycle. It has been previously suggested that these are resultant from the fusion of the outer membranes of DMVs^4,30^ and speculated that the fusion of DMVs into VPs is necessary to repurpose membranes for budding viruses^4^. We have not observed assembly intermediates or budding viruses in DMV or VP membranes. Until very recently, DMVs were thought to be completely enclosed, which raised the question of how the viral mRNAs could gain access to the cytoplasm to be translated. We have observed several double-membrane-spanning pore complexes in DMVs (Fig. 2B–D, yellow arrow), resembling the RNA transport portal seen in DMVs of MHV- or SARS-CoV-2-infected cells^27^. However, such portals appear less frequently in our dataset (extrapolated average of 0.5 portals per DMV, total 40 portals from 114 milled DMVs) compared to the number previously reported for MHV (extrapolated average of 8 portals per DMV)^27^.

### SARS-CoV-2 assembly and budding

**Fig. 3:**
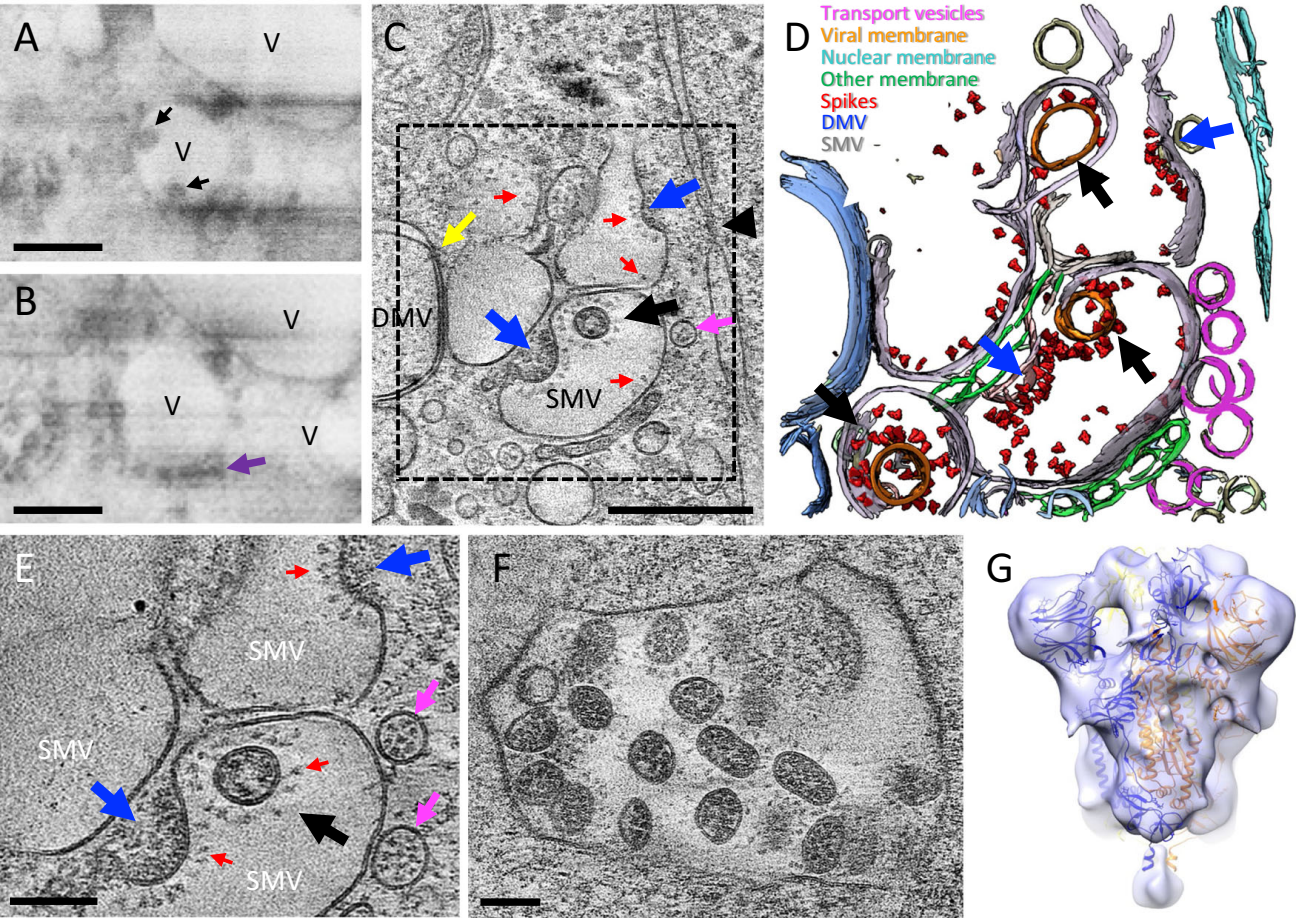
SARS-CoV-2 cytoplasmic viral assembly. **A**, **B** CryoFIB/SEM images of two sequential slices separated by 80 nm. Black arrows point to virus particles in a vesicle (V). Purple arrow points to small electron-dense material lining the outside of virus-containing vesicle. **C** Tomographic slice of cryoFIB lamella depicting SARS-CoV-2 assembly, with DMV portals (yellow arrow), assembling viruses (blue arrow), assembled virus (black arrow), viral spikes on SMV membranes (red arrows), transporting vesicles around the assembly site (pink arrow), and a nucleopore (black arrowhead). **D** Density segmentation of **C**, displaying three virus particles (black arrows) and two assembly sites (blue arrows). **E** An enlarged view (at a different angle) of boxed area in **C**, showing assembled virus (black arrow), assembling viruses (blue arrows), spikes (red arrows), and spike-containing vesicles (pink arrows). **F** Large intracellular virus-containing vesicle (LVCV) full of readily assembled viruses. **G** Subtomogram average of viral spikes of intracellular viruses from cell lamellae at 16 Å resolution, fitted with an atomic model of spike trimer (PDB 6VXX)^16^. Scale bar is 300 nm in **A**–**C** and 100 nm in **E**, **F**.

The translation of the subgenomic vRNAs gives rise, among others, to the structural proteins N, M, E, and S, which are required for assembly. M, E, and S are membrane-associated proteins and are localized to the ER, Golgi, and the ERGIC^31,45^. The N protein associates with the genomic vRNA and M protein, which is thought to drive vRNA packaging and genome encapsidation^46,47^. The main assembly and budding site of other coronaviruses has been previously described at the ERGIC by conventional EM of stained plastic sections^8,29,31,45^. In serial cryoFIB/SEM images of SARS-CoV-2-infected cells, we observed vesicles containing virus particles (Fig. 3A, black arrows), with an array of small dense material (Fig. 3B) lining along the vesicle membrane. The similar architecture was captured by high-resolution cryoET of cell lamella, which shows that these single membrane vesicles (SMVs) are SARS-CoV-2 assembly and budding sites (Fig. 3C–E). Next to SMVs, there are small vesicles containing discrete densities (Fig. 3C–E, pink arrows). CryoET and subtomogram averaging further revealed that these small vesicles are SARS-CoV-2 spike containing transport vesicles (Supplementary Fig. 5), possibly supplying newly synthesized spikes and other viral components via fusion with SMVs where viral assembly takes place (Fig. 3C–E, pink arrows, and Supplementary Movie 8). Indeed, spikes are observed on SMV membranes sparsely distributed or otherwise clustered at the assembly sites (Figs. 2C and 3C–E (red arrows) and Supplementary Movie 8). Interestingly, several SARS-CoV-2 assembly intermediates were observed within a single tomogram from a cell lamella (Fig. 3C–E, blue arrows, and Supplementary Movie 8), along with fully assembled virus particles released into SMVs (Fig. 3C–E, black arrows, and Supplementary Movie 8), thus capturing the assembly and budding process of SARS-CoV-2. It is conceivable that, upon fusion of transport vesicles with the SMV, spikes are readily diffused on the SMV membrane. They cluster when interacting with N-associated vRNA, possibly via M protein^20,46^, which initiates the assembly and budding process that finally releases the viral particle into the SMV. Consistent with this, spike clusters are observed exclusively associated with the agglutination/gathering of electron-dense material, which presumably represents viral genome (Fig. 2C, blue arrow). Noticeably, the virus assembly site is frequently present in the vicinity of RNA portals in DMVs (Fig. 2B–D, yellow arrows), potentially facilitating the assembly process.

**Fig. 4:**
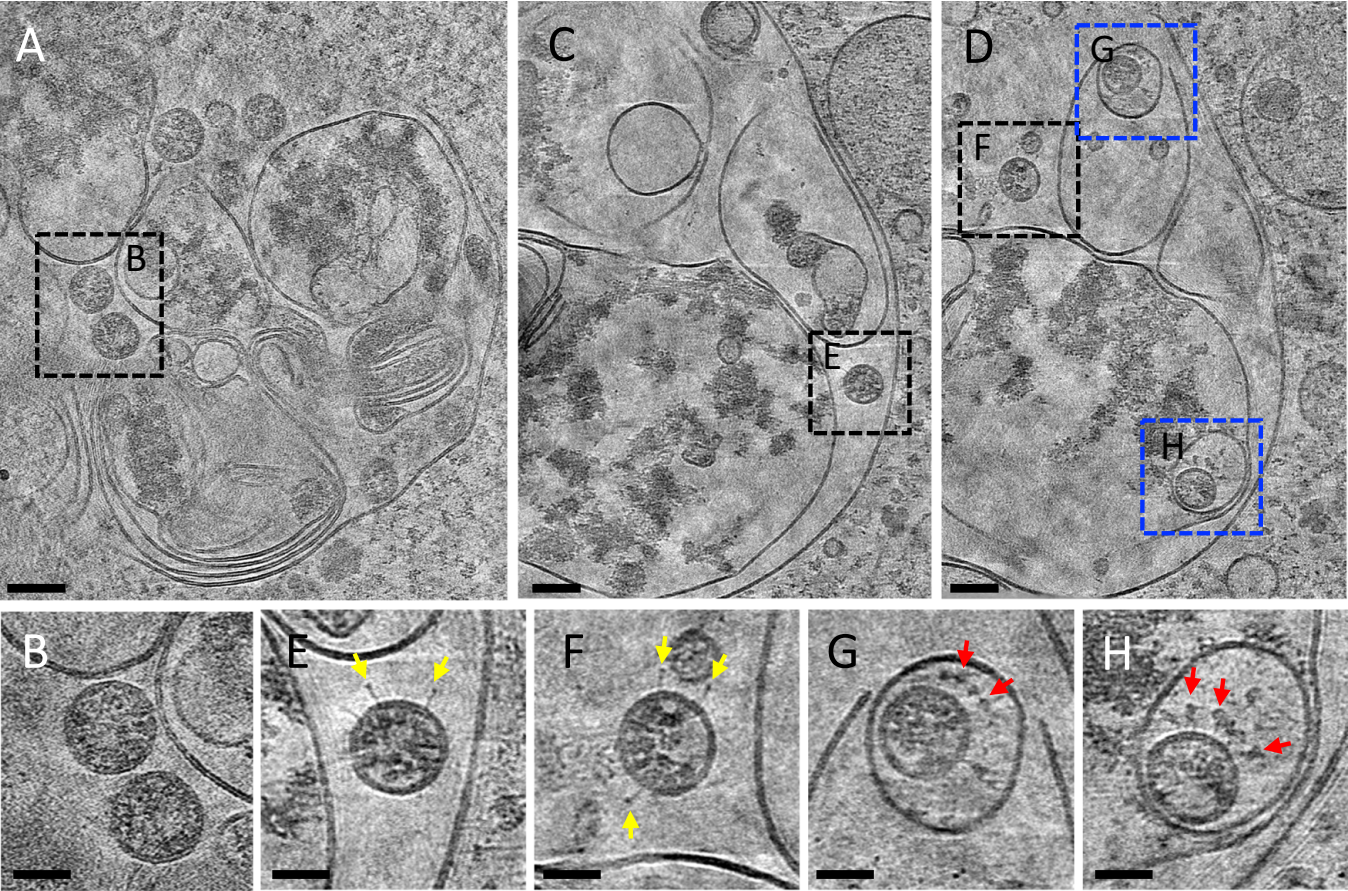
Viruses in complex membrane compartments (CMCs). **A** A tomographic slice of a CMC in cell lamella depicting convoluted membranes containing virus particles (boxed area). **B** Detailed tomogram slice of spikeless viruses from the boxed area in **A**. **C**, **D** Consecutive tomographic slices of the same CMC separated by 140 nm, containing viruses (black and blue boxed areas). **E**, **F** Detailed tomogram slice of viruses with a few postfusion spikes (yellow arrows) from boxed areas in **C**, **D**. **G**, **H** Detailed tomogram slice of viruses protected by single membrane vesicles (SMVs) harboring prefusion spikes (red arrows) from blue boxed areas in **D**. Scale bars are 100 nm in **A**, **C**, **D**; 50 nm in **B**, **E**–**H**.

Most virus particles are found in SMVs, which contain single or multiple virions (Fig. 3E, F). CryoET and subtomogram averaging of 856 spikes from these particles yielded a density map at 16 Å resolution (at 0.143 FSC cut-off) by emClarity^48^ (Fig. 3G and Supplementary Fig. 6). The averaged density map resolves the overall spike structure, which overlaps well with prefusion spike atomic models^13–17^ (Fig. 3G). Some virus particles were also observed in electron-dense complex membrane compartments (CMCs) (Fig. 4, dashed boxes). There were two types of virus particles: viruses protected by SMVs in CMCs show prefusion spikes (Fig. 4D, G, H); viruses in the lumen of CMCs, however, have either no spikes (Fig. 4B) or a few postfusion spikes on their surfaces (Fig. 4E, F). The fact that the spike proteins are in the postfusion state suggests that proteolytic processing has taken place in these compartments resulting in S1 shedding. The presence of postfusion spikes in intracellular viruses might be an indication that these virus particles could be off-pathway viral assemblies. Alternatively, as our samples were cryofixed at 24 hpi, re-infection is also possible and these structures may represent remnants of late endosomes from viral entry or lysosomes for viral degradation. It has been recently proposed that beta-coronaviruses explore the lysosomal pathway to exit the cell^32^. Although it is difficult to confirm the nature of the CMCs by cryoEM alone, they are morphologically similar to lysosomal residual bodies^32^. It is possible that the particles present in SMVs inside CMCs are infectious. However, in our study we observed only two instances of SMV-protected viruses in CMCs, compared to the large number of virus particles in LVCVs, suggesting that this is unlikely to represent a major pathway for virus egress.

### SARS-CoV-2 egress

**Fig. 5:**
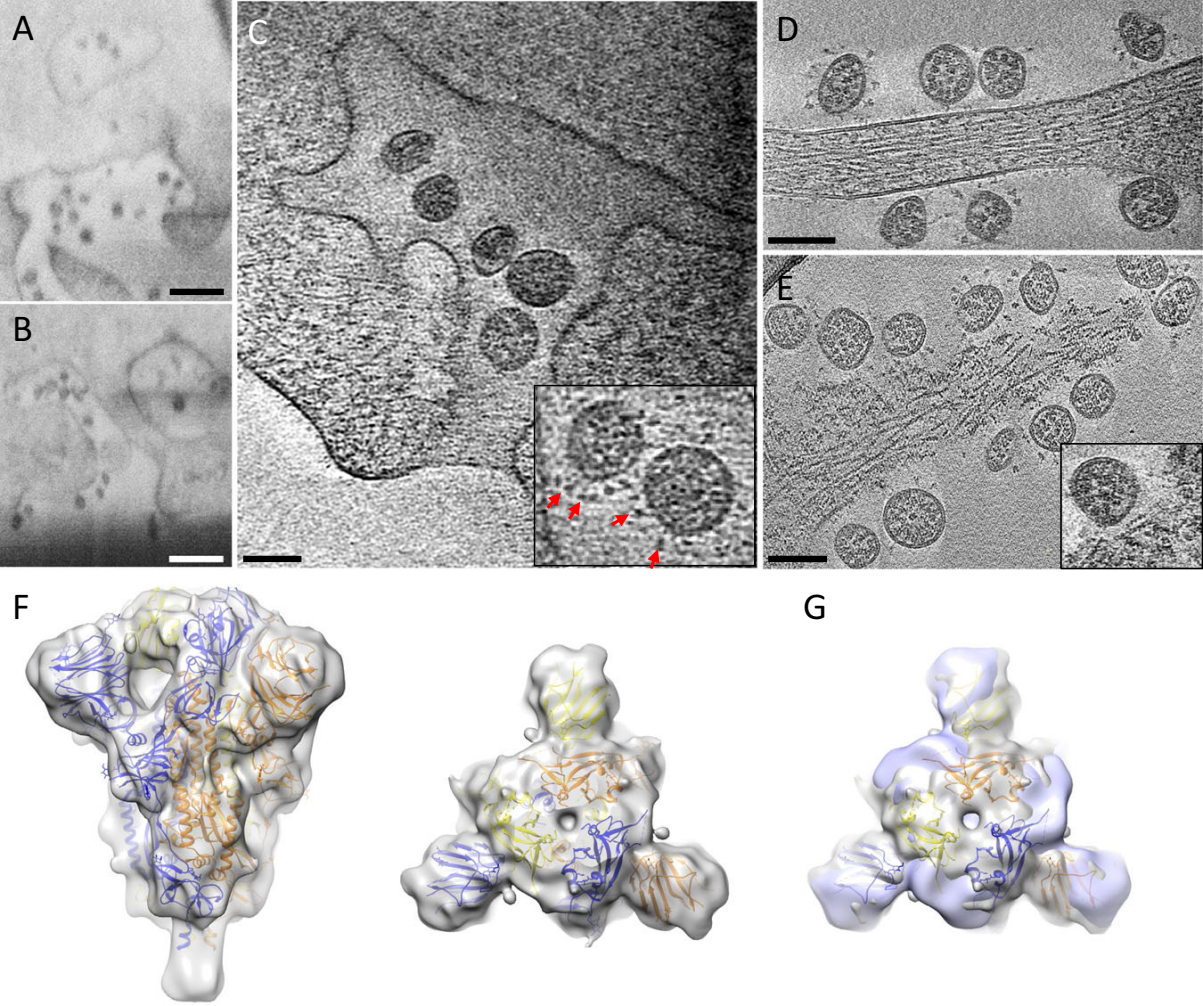
SARS-CoV-2 egress. **A**, **B** CryoFIB/SEM images of cell periphery, depicting virus particles exiting through extended tunnels connected to external of the cell. **C** CryoET of the SARS-CoV-2-exiting tunnel. Inset shows a close-up view of viruses with prefusion spikes (red arrows). **D**, **E** Viruses released from the cell, close to an intact cell membrane (**D**) or next to membrane lesion sites (**E**). Inset, close-up view of a membrane lesion site. **F** Subtomogram average of spikes on released viruses at 10.6 Å resolution fitted with an atomic model of spike trimer (PDB 6VXX)^16^, viewed from side and top. **G** Comparison of spike structures from intracellular assembled viruses (transparent blue) and extracellular released viruses (transparent gray), viewed from top. Scale bar is 300 nm in **A**, **B** and 100 nm in **C**–**E**.

Previous analyses on how SARS-CoV-2 viruses are released from cells were mainly by fluorescent light microscopy^32^ and conventional EM imaging of stained plastic-embedded samples^32,36^. There has not been detailed study using high-resolution cryoEM/ET. We investigated SARS-CoV-2 egress using both serial cryoFIB/SEM volume imaging and cryoET under frozen-hydrated conditions. CryoFIB/SEM images reveal viruses exiting tunnels in 3D at the cell periphery connecting to cell membrane (Fig. 5A, B and Supplementary Movie 2). This likely resulted from the fusion of large multi-virus-containing vesicles with cell membrane, i.e., egress through exocytosis-like mechanism. Consistent with cryoFIB/SEM analysis, we also observed virus-exiting tunnels in cryo-tomograms (Fig. 5C). Given that these compartments often contained many viral particles with prefusion spikes, it is likely a snapshot of viral exit, although macropinocytosis-facilitated entry is still conceivable^49^.

In addition, we also frequently found plasma membrane discontinuities, or membrane lesions, in tomograms recorded on the infected cell periphery (Fig. 5E and Supplementary Movie 9). In all, 44.6% of tomograms from infected cells show cell membrane lesions (total of 116 membrane lesion sites in 74 tomograms), whereas 18.7% tomograms from uninfected cells display similar but more sparse membrane lesions (10 membrane lesion sites from 16 tomograms, *p* = 0.089, Supplementary Fig. 7A). The membrane lesions are discrete in appearance, with a slightly smaller lesion diameter in infected cells than in uninfected controls (50.21 ± 14.11 nm in infected cells and 68.05 ± 16.74 nm in control cells, *p* = 0.01, Supplementary Fig. 7B). Over 72% of the membrane lesions in infected cells had at least one extracellular virus particle nearby (average distance between lesion and virus = 77.73 ± 18.67 nm, *n* = 84, Supplementary Fig. 7C). Close inspection of individual membrane lesions indicates that the underlying cytoskeleton, such as actin filaments, appears retained (Fig. 5E). These lesions could be an indication of membrane fragility due to infection. Similar lesions have been previously observed on SARS-infected cells using atomic force microscopy^50^.

CryoET subtomogram averaging of 2179 spikes from extracellular virus particles (Supplementary Movie 10) yielded a density map at 10.6 Å resolution (at 0.143 FSC cut-off), which represents the prefusion state (Fig. 5F and Supplementary Fig. 6A). It is very similar to the spike structure derived from purified virus particles (Supplementary Fig. 6B)^48^. Comparison between the spike structures from intracellular assembled and extracellular released virus particles showed similar prefusion architecture with a small difference in the orientation of N-terminal and receptor-binding domains (Fig. 5G), suggesting that no further major structure rearrangement takes place for viral spikes from assembly to egress, given the current resolution. Previously, spike structures have mainly been derived in vitro from either recombinant proteins or from purified inactivated virus particles^11–17,33–35^. The two spike structures presented here were derived directly from infected cells in cellular context and are thus a close representation of the native state.

## Discussion

**Fig. 6:**
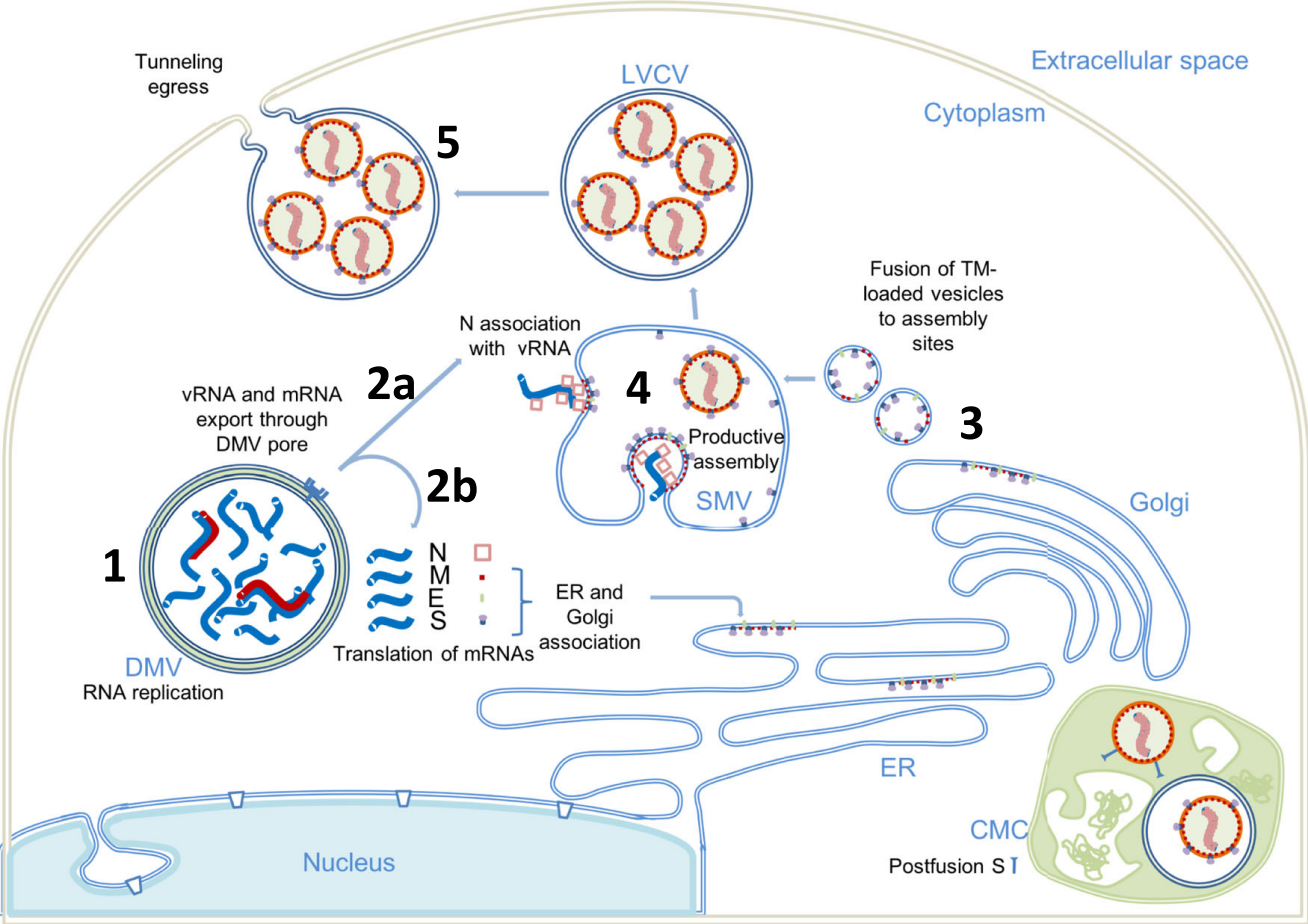
A plausible model of SARS-CoV-2 replication. (1) Viral genome replication occurs inside the DMVs, generating the negative-strand viral and subgenomic RNAs (red), positive vRNA genomic copy, and subgenomic mRNAs (blue). (2) Positive RNAs are exported to cytoplasm through the DMV pores. Subgenomic mRNAs are translated (2b). Structural proteins M, E, and S associate with ER and Golgi membranes. Genomic vRNA becomes complexed with newly synthetized N (2a). (3) S, E, and M are transported in dense vesicles, which are fused with SMVs. (4) Productive viral assembly happens in the SMV clustering the viral spikes and encapsidating the genome in RNPs. Viruses bud to the internal space of the SMV. (5) Egress occurs through tunnels. The complex membrane compartment (CMC) is depicted in green, which encloses both postfusion spike-containing virus particles and prefusion spike-containing virus particles. DMV double membrane vesicle, SMV single membrane vesicle, CMC complex membrane compartment, LVCV large virus-containing vesicle.

We used a correlative approach to image the SARS-CoV-2 infection in near-native vitrified cells, encompassing multiple spatial scales, from the whole-cell level to the subcellular and molecular levels. This approach yielded a holistic view of the infection process describing virus-induced cytopathies and their correlation to in situ structures. Crucially, the integration of multi-scale imaging data achieved through this workflow (Supplementary Fig. 1) has allowed us to directly visualize structural events of SARS-CoV-2 replication in the context of the cytopathic consequences in the infected cell. Given prior knowledge of SARS-CoV-2 and other coronaviruses from previous studies^4,7,9,29^, these images led us to propose an enhanced model for SARS-CoV-2 replication, in particular virus genome replication, assembly, and egress, described below. Images of the replication process of SARS-CoV-2 reveal it to be a spatially well-organized and highly efficient process. From genome replication to protein synthesis and transport to virus assembly and budding, each step takes place in purpose-specific close-knit cytoplasmic compartments. As illustrated in Fig. 6, RNA replication, including genomic vRNA and subgenomic mRNA, occurs in DMVs, secluding them from the host cell innate immune response (step 1). The newly synthesized vRNAs are then transported out of DMVs through transmembrane portals to virus assembly sites proximal to these DMVs (step 2a), whereas mRNAs exit through the same portals to the cytoplasm and subsequently translocate to the ER/Golgi for protein production (step 2b). The viral spikes, in a trimeric prefusion form produced in ER/Golgi networks, are transported to assembly sites via small transport vesicles (step 3). These vesicles fuse with SMV membranes where viral spikes cluster at the assembly site in the presence of vRNA and N protein resulting in a positive membrane curvature and finally budding of virions into the SMV (step 4). The assembly and budding processes give rise to virus-containing vesicles, which can contain multiple virus particles. Virus particles can then exit through tunnels, maybe by lysosomal exocytosis^32^ (step 5). Aspects of this model remain speculative and will require further experimental validation in future studies.

A multi-scale view of the processes of genome replication, assembly, and egress of the SARS-CoV-2 virus during its journey through the cell is critical in advancing our understanding of this pathogen as it bears the means to elucidate new information and inspire medical interventions to stop productive infections. There are many facets of this process awaiting further investigation to dissect the molecular mechanism of SARS-CoV-2 replication, including the roles of other viral proteins, such as M and E, as well as host proteins and machines. This study provides a direct look of the SARS-CoV-2 replication cycle under near-native conditions and structures of prefusion spikes directly from cellular assembled and extracellular released virus particles. The unique methodologies and workflow developed through this study can be broadly applied to studies of infection processes of other human pathogens beyond SARS-CoV-2.

## Methods

### Cell lines and viruses

African green monkey kidney Vero Ccl-81 cells (Female) (ATCC, CCL-81) were maintained in Dulbecco Modified Eagle media supplemented with 5% fetal bovine serum, 10 units/mL penicillin (Gibco), 10 µg/mL streptomycin (Gibco), and 2 mM L-glutamine. Cell line has not been authenticated.

SARS-CoV-2 isolate BetaCoV/England/02/2020 (EPI_ISL_407073) was deposited by Professor Maria Zambon and obtained through BEI Resources, NIAID, NIH: SARS-Related Coronavirus 2, Isolate England/02/2020, NR-52359. The viral stock used in this study has been titrated by plaque assay in Vero Ccl-81 cells and sequenced to confirm retention of the furin cleavage site through the amplifying passages.

### Sample preparation

Vero Ccl-81 cells (ATCC) were maintained as above. Sixteen thousand cells were seeded on the carbon-side of fibronectin-treated G300F1 R2/2 gold EM grids in a six-well plate well. Infections were performed using passage 3 of SARS-CoV-2 England/02/2020 at MOI of 0.5, 0.1, or 0 (for negative controls). Media was removed from the Vero Ccl-81 cells (ATCC) and replaced with an appropriate amount of virus diluted in 0.5 mL of Dulbecco’s modified Eagle medium (Merck) with 1% fetal calf serum, 10 units/mL penicillin (Gibco), 10 µg/mL streptomycin (Gibco), and 2 mM L-glutamine (Gibco). The cells were incubated at room temperature for 15 min after which a further 1.5 mL of media was added to each well. The plate was then incubated at 37 °C for 24 h following which supernatants were discarded and cells washed with 2 mL of PBS. The cells were then fixed by addition of 3 mL of 4% paraformaldehyde in PBS for 1 h at room temperature. After fixation, grids were placed in a Leica grid plunger GP2, 1 µl of concentrated 10 nm gold fiducials in BSA (Electron Microscopy Sciences) was applied to the back-side of the EM grid and blotted from the back-side and the grid was plunge frozen in liquid ethane after blotting. Vitrified grids were stored in liquid nitrogen until data collection.

### CryoET data acquisition

Tilt series acquisition was carried out at a FEI Titan Krios G2 (Thermo Fisher Scientific) electron microscope operated at 300 kV and equipped with a Gatan BioQuantum energy filter and post-GIF K3 detector (Gatan, Pleasanton, CA). Tilt series were recorded using SerialEM version 3.8 tilt series controller with pixel sizes of 1.63, 2.13, and 4.58 Å for intact cells and 2.13 and 7.58 Å on lamella. Zero-loss imaging was used for all tilt series with a 20 eV slit width. Defocus values ranged from −2 to −7 µm, except for lamella at 7.58 Å pixel size where 50 µm defocus was used. A 100 µm objective aperture was inserted. A grouped dose-symmetric scheme was used for all tilt series; intact cells were collected with a range of +/−60 degrees at 3 degree increments in groups of 3 and total dose of 120–135 e/Å^2^; lamella with +/−54 degrees at 3 degree increments and groups of 3 with total dose of 120–135 e/Å^2^ at 4.58 Å and +/−54 degrees at 3 degree increments and groups of 10 with total dose of 70–90 e/Å^2^ at 7.58 Å. Autofocus and tracking was performed at each tilt with drift measurement taken at tilt reversals with a 10 Å/s target rate. At each tilt, 5 movie frames were recorded using correlated double sampling in super-resolution mode and saved in lzw compressed tif format with no gain normalization. Movies were subsequently gain normalized during motion correction and Fourier cropped back to physical pixel size. After each tilt series, a script was run to take a fresh dark reference and reset the defocus offset. A total of 294 tilt series were collected, of which 56 tilt series were from control uninfected cells (20 from cell lamella and 36 from cell periphery) and 238 tilt series were from SARS-CoV-2-infected cells (90 from lamella and 148 from cell periphery).

### CryoFIB lamella preparation

Lamella milling of SARS-Cov-2-infected cells was carried out using a Scios DualBeam cryoFIB (ThermoFisher Scientific) equipped with a PP3010T transfer system and stage (Quorum Technologies) using the xT v7.6 software (ThermoFisher Scientific). Grids were sputter coated within the PP3010T transfer chamber maintained at −175 °C. After loading onto the Scios stage at −168 °C, the grids were inspected using the SEM (operated at 5 kV and 13 pA) and infected cells identified by correlation from transmission electron microscopy (TEM). The grid surface was coated using the gas injection system (Trimethyl(methylcyclopentadienyl)platinum(IV), ThermoFisher Scientific) for 3 s, yielding a thickness of ~3 µm. Milling was performed using the ion beam operated at 30 kV and currents decreasing from 300 to 30 pA. At 30 pA, lamella thickness was <300 nm. During the final stage of milling, SEM inspection of the lamellae was conducted at 2 kV and 13 pA.

### CryoET image processing

The frames in each tilt angle in a tilt series were processed to correct drift using MotionCor2^51^. For the intact cell dataset, tilt series were aligned using the default parameters in IMOD version 4.10.22 with the eTomo interface, using gold-fiducial markers^52^. For lamella dataset, tilt series were aligned in the framework of Appion-Protomo v1.2.2 fiducial-less tilt-series alignment suite^53^. After tilt series alignment, the tilt series stacks together with the files describing the projection transformation and fitted tilt angles were transferred to emClarity 1.5.3.03 for the subsequent subtomogram averaging analysis^48^.

### Subtomogram averaging

All subtomogram averaging analysis steps were performed using emClarity 1.5.3.03, mostly following previously published protocols described workflow^48^. In summary, CTF estimation for each tilt was performed by using emClarity. Particle picking was carried out using the template matching function within emClarity using reference derived from EMDB-21452^16^. The template and tomogram are band-pass filtered to the first CTF-zero in the tomogram, determined in the previous CTF estimation step, and 600 Å, to remove eventual ice gradient. The filter is smoothed by a Gaussian roll-off at the 600 Å boundary and a cosine edge at the low-resolution boundary. The template matching results were cleaned manually by comparison of the binned tomograms overlaid with the emClarity-generated IMOD model showing the *x*,*y*,*z* coordinates of each cross-correlation peak detected. After manually template cleaning, a total of 856 subvolumes from the lamella dataset and a total of 2179 subvolumes from the extracellular viruses dataset were retained, deriving from 2 tilt series and 10 tilt series respectively, for the following averaging and alignment steps in emClarity. For the extracellular viruses dataset, the 3D iterative averaging and alignment procedures were carried out gradually with binning of 3×, 2×, 1×, each with 2–3 iterations with increasingly restrictive search angles and translational shifts. Threefold symmetry was applied during all the steps. Final converged average map was generated using bin1 tomograms with pixel size of 1.63 Å/pixel and a box size of 222 × 222 × 222 voxels. Resolution indicated by 0.143 FSC cut-off was 10.6 Å. A map–map FSC comparison was performed between the final averaged map and the high-resolution subtomogram average of the SARS-CoV-2 spike from purified virions EMD-11222^34^ using EMAN2.2^54^. The calculated FSC at 0.143 threshold was 10.8 Å. The same averaging and aligning process was carried out for lamella dataset, except for the final average map was generated with pixel size of 2.13 Å/pixel and a box size of 177 × 177 × 177 voxels and a final resolution at 16 Å (0.143 FSC cut-off). For the spikes inside transport vesicles, particle picking was carried out using the template matching function within emClarity using reference derived from EMDB-21452^16^. The template and tomogram were band-pass filtered to 50–600 Å. Forty-two subtomograms were iteratively aligned and averaged through 6 iterations (the first 4 with 4× binning, the last 2 with 3× binning). Threefold symmetry was applied during all the steps. Final converged average map was generated using bin3 tomograms with pixel size of 6.39 Å/pixel and a box size of 72 × 72 × 72 voxels. Resolution indicated by 0.143 FSC cut-off was 24.5 Å.

### Serial cryoFIB/SEM volume imaging

Samples were imaged on a Zeiss Crossbeam 550XL fitted with a Quorum transfer station and cryo-stage. They were mounted on a Quorum-compatible custom sample holder and coated with platinum for 60 s at 10 mA on the Quorum transfer stage, prior to loading on the cryo-stage. Stage temperature was set at −165 °C, while the anticontaminator was held at −185 °C. Samples were imaged at 45° tilt after being coated again with Pt for 2× 30 s using the FIB-SEM’s internal GIS system, with the Pt reservoir held at 25 °C. Initial trapezoid trenches were milled at 30 kV, 7 nA over 15 μm to reach a final depth of 10 μm, with a polish step over a rectangular box with a depth of 10 μm performed at 30 kV, 1.5 nA. Serial sectioning and imaging acquisition was performed as follows: FIB milling was set up using the 30 kV, 700 pA probe, a *z*-slice step of 20 nm, and a depth of 10 μm over the entire milling box; SEM imaging was performed at a pixel depth of 3024 × 2304 pixels, which resulted in a pixel size of 6.5 nm, with the beam set at 2 kV, 35pA, dwell time 100 ns, and scan speed 1, averaging the signal over 100 line scans as a noise-reduction strategy. Software versions used were Zeiss SmartSEM v6.06 and Zeiss SmartFIB v1.11.10 (Zeiss).

### Serial cryoFIB/SEM Segmentation

Cell structures were manually segmented from stacks of images using ImageJ 1.8.0_172^55^ and Microscopy Image Browser (MIB) software version 2.70^56^ on a Windows computer with 32 GB RAM and Wacom Cintiq Pro display tablet with pen. Datasets of <2 GB in .mrc format were analyzed one at a time, where one dataset comprised of 200 subsequent images on average.

### CryoET segmentation and 3D visualization

Transport vesicles, viral membrane, nuclear membrane, DMVs, and SMVs were segmented using convolutional neural network-based tomogram annotation in the EMAN2.2 software package^57^. Viral spikes were mapped back to their original particles position using emClarity tomoCPR function. UCSF Chimera v1.13^58^ was used to visualize the segmentations and subtomogram average structures in 3D.

### Soft X-ray cryo-tomography

Data were collected in areas of interest on vitrified cells grown on 3 mm TEM grids according to established protocols^39^. Samples were loaded on the X-ray microscope at B24 and were first mapped using visible light with a ×20 objective. The resulting coordinate map was used to locate areas of interest where two-dimensional X-ray mosaics were collected (X-ray light used was at 500 eV) and used to identify areas of interest within. Tilt series of 100–120° were collected in XRM Data Explorer 13.1.9050.40704 (Carl Zeiss X-ray microscopy) for each field of view area of interest at 0.2 or 0.5° steps with constant exposure of 0.5 s keeping average pixel intensity to between 5 and 30k counts (10 nm/pixel, FOV 10 × 10 μ). All tilt series were background subtracted, saved as raw tiff stacks, and reconstructed using either manually in IMOD 4.10.22^52^ or using IMOD Batchruntomo automated reconstruction^59^.

### Quantification and statistical analyses

Statistical analyses were performed in GraphPad Prism 9. Number of portals in DMV and plasma membrane discontinuities were determined after visual inspection and manual counting by two independent investigators. Two-tailed Fisher exact test was used to compare the number of membrane lesions in infected and uninfected cells. To compare the sizes of membrane lesions, an unpaired Welch’s *t* test was used. Quantification of mitochondria numbers in soft X-ray tomograms was determined by visual inspection and manual counting by one investigator. The number of mitochondria was normalized by the cytoplasmic area of the tomogram (after deducting areas of nucleus and outside the cell). Welch’s *t* test was used to compare mitochondria numbers from infected and uninfected cells. For serial cryoFIB/SEM volumes, quantification of mitochondria end-point sizes and volume was performed by manual segmentation of the whole-cell mitochondria network in infected and uninfected cells in serial cryoFIB/SEM volumes by three investigators. Welch’s *t* test was used to compare mitochondria size and volume between infected and uninfected cells. The investigators were not blinded during experiments and outcome assessment.

### Reporting summary

Further information on research design is available in the Nature Research Reporting Summary linked to this article.

## Supplementary information

Supplementary infomation

Supplementary Movie 1

Supplementary Movie 2

Supplementary Movie 3

Supplementary Movie 4

Supplementary Movie 5

Supplementary Movie 6

Supplementary Movie 7

Supplementary Movie 8

Supplementary Movie 9

Supplementary Movie 10

Reporting Summary

## Data Availability

The previously published atomic model and EM map of the SARS-CoV-2 prefusion spike have accession codes PDB6VXX and EMD-21452, respectively^16^. The in situ SARS-CoV-2 spike subtomogram averaged map used for map–map FSC comparison has accession code EMD-11222^34^. The raw cryoET tilt series of SARS-CoV-2-infected cells generated in this study are deposited at EMPIAR under accession code EMD-10753. The cryoEM density maps for prefusion SARS-CoV-2 Spike Protein from intracellular assembled virions and those released from cells generated in this study have been deposited at the Electron Microscopy Database under accession code EMD-12992 and EMD-12991, respectively.
