# Supplementary material for: Correlative multi-scale cryo-imaging unveils SARS-CoV-2 assembly and egress
Source: Nat Commun. 2021 Jul 30;12:4629. doi: 10.1038/s41467-021-24887-y (PMC8324836; doi:10.1038/s41467-021-24887-y)
Supplement: Supplementary file 1 — Supplementary infomation [file 41467_2021_24887_MOESM1_ESM.pdf]

Supplementary Table 1 | Details of analyzed samples and summary of quantification on biological findings.

	SARS-CoV-2 infected	Uninfected Control
**Total number of data sets**		
Imaged Grids in this study	14	4
CryoET		
Cryo-tomograms (total)	238	56
Cell Lamella tomograms	90 (4 milled grids)	20 (1 milled grid)
Cell periphery tomograms	148	36
Soft X-ray tomography		
Cells imaged	5 (infection confirmed by cryoET)	12
Serial cryoFIB/SEM		
Cells imaged	5 (infected cells, displaying viral particles in cell periphery)	6
**Frequency of events**		
DMVs	114 (from 24 cryo-tomograms)	n/a
Portals	40 (0.35 portal/DMV)	n/a
Tomograms with membrane lesions (%) (*p*=0.089)*	44.6% (33 of 74 periphery tomograms)	18.7% (3 of 16 periphery tomograms)
Membrane lesion sites	116 (in 74 tomograms, from 5 different grids)	10 (in 16 tomograms, from 1 grid)
Lesion diameter (*p*=0.01)**§	50.21 ± 14.11 nm	68.05 ± 16.74
Distance from lesion to closest virus§	77.73 ± 18.67 nm	n/a
Mitochondria number	76 (in 8 soft X-ray tomograms)	448 (in 9 soft X-ray tomograms)
Average number of mitochondria/tomogram (*p*=0.0085)**§	18.2 ± 10.7	95.5 ± 28.5
Mitochondria length (*p*=0.001)** §	0.60 ± 0.70 μm (in 3 manually segmented serial cryoFIB/SEM imaged cells)	0.90 ± 1.14 μm (in 2 manually segmented serial cryoFIB/SEM imaged cells)
Mitochondria volume (p=0.113)** §	0.058 ± 0.196 μm^3 (in 3 manually segmented serial cryoFIB/SEM imaged cells)	0.142 ± 0.764 μm^3 (in 2 manually segmented serial cryoFIB/SEM imaged cells)
**Subtomogram averaging**		
Intracellular spikes	856	n/a
Extracellular spikes	2179	n/a

* Fisher exact test, two tailed

** Welsh corrected t test, unpaired, two tailed

§ Mean and standard deviation

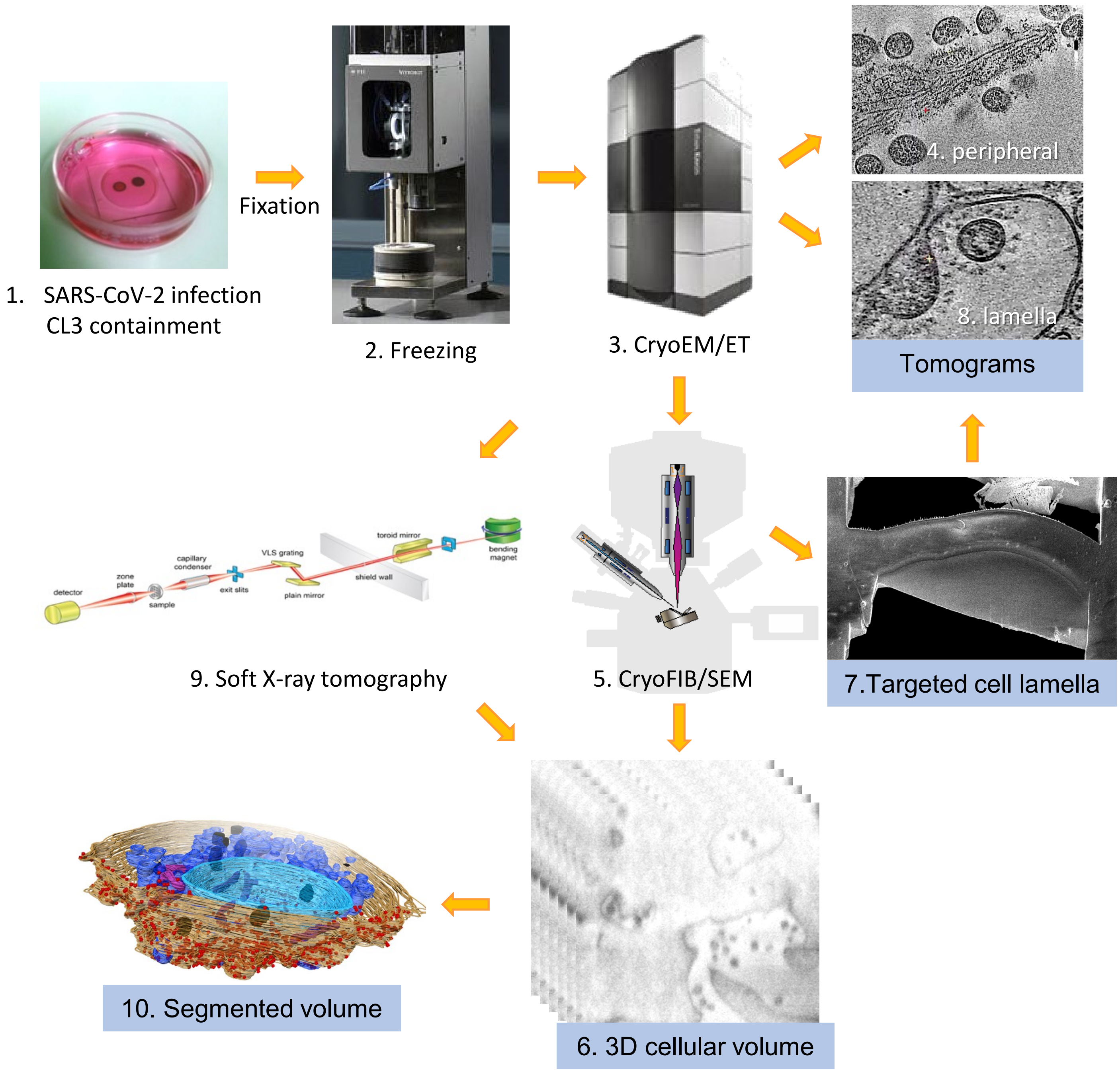

Supplementary Figure 1 | A workflow for correlative multi-modal multi-scale imaging of SARS-CoV-2 infected cells. 1) Cells are grown on indexed EM grids, infected with SARS-CoV-2 and fixed with paraformaldehyde. 2) Grids are plunge-frozen in liquid ethane and 3) imaged by cryoEM/ET to locate the infected cells. 4) Tomograms are collected on the cell periphery of infected cells. 5) Infected cells are subjected to processing and imaging in a cryoFIB/SEM dualbeam instrument for 6) serial cryoFIB/SEM volume imaging and 7) targeted cell lamella. 8) Tomograms are collected from cell lamellae. 9) Alternatively, infected cells are imaged by soft-X-ray cryo-tomography. 10) Cellular volume data are manually segmented.

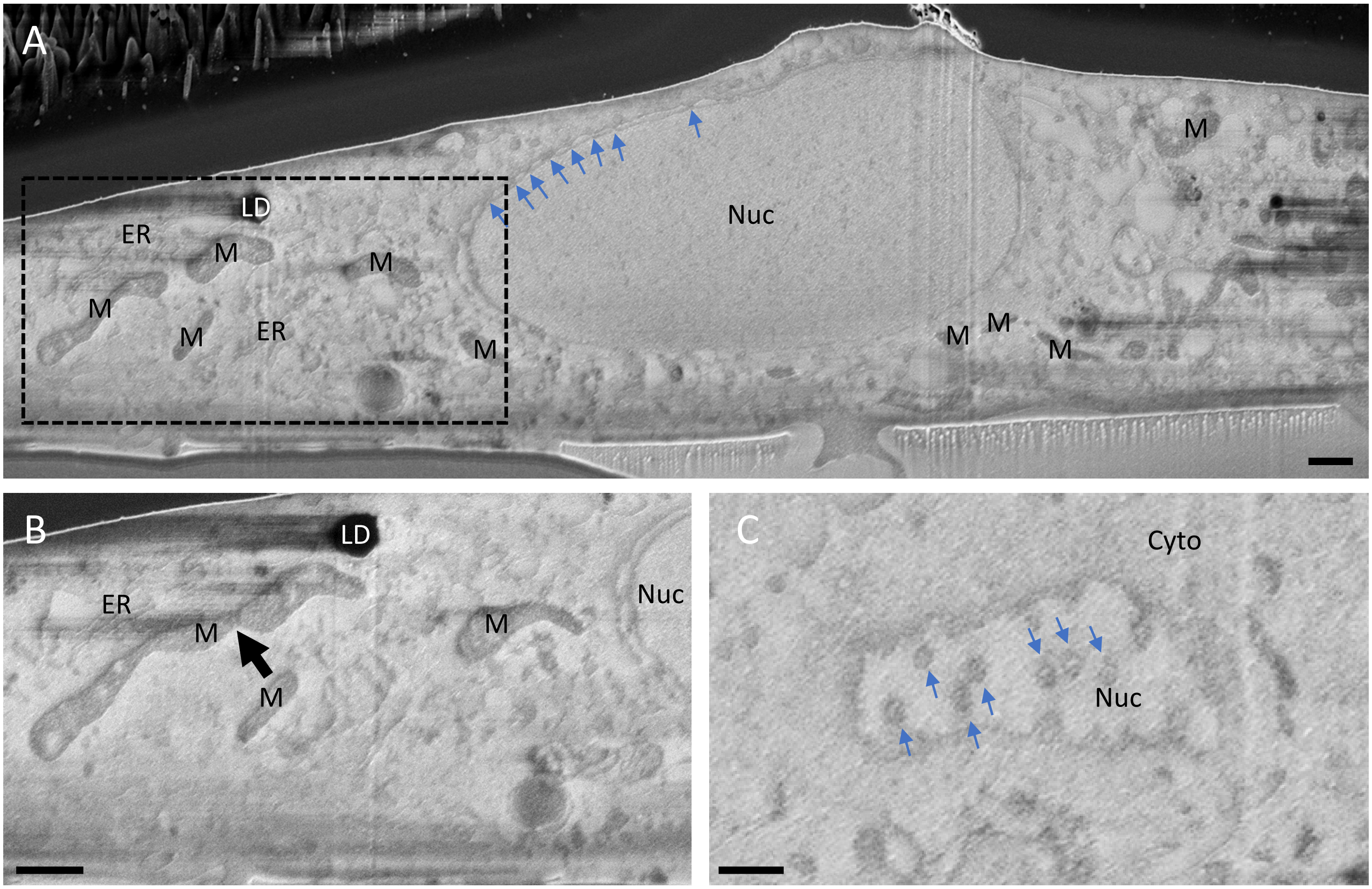

Supplementary Figure 2 | Serial cryoFIB/SEM of control uninfected cell. (A) A representative overview of a cryoFIB/SEM slice from an uninfected cell. Blue arrows point to nuclear pores. (B) Detailed view of the dashed area in A from a slice 100 nm in depth depicting a mitochondrial network. (C) A cryoFIB/SEM slice tangential to the nuclear envelope, showing top view of nucleopores (blue arrows). Nuc, nucleus; Cyto, cytoplasm; ER, endoplasmic reticulum; M, mitochondria; LD, lipid droplet. Scale bars, 300 nm. Representative of 6 uninfected cells imaged by serial cryoFIB/SEM.

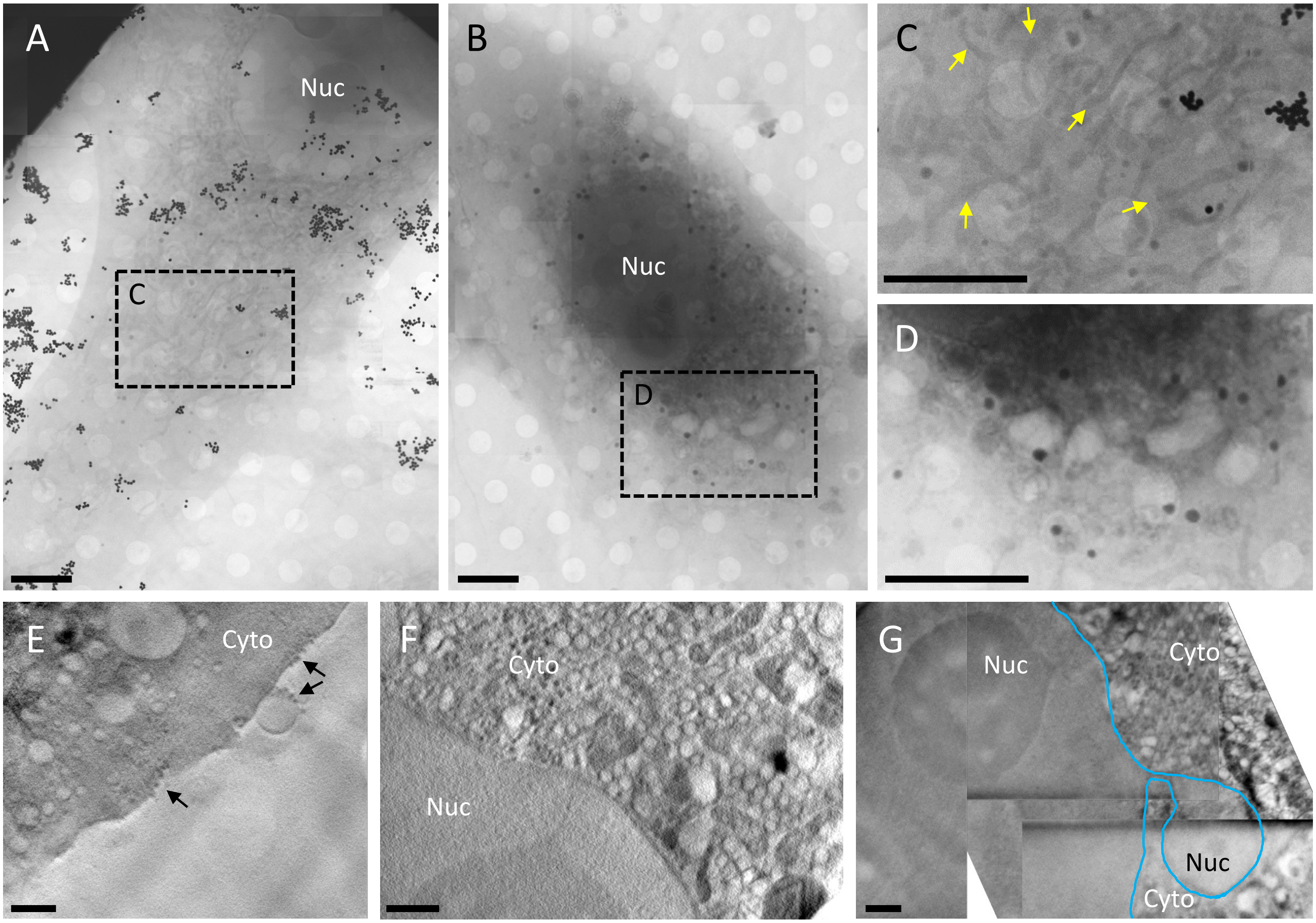

Supplementary Figure 3 | Soft X-ray cryo-tomography of SARS-CoV-2 infected cells. (A-B) Soft X-ray overview mosaics of uninfected (A) and infected (B) Vero cells. (C-D) Detailed view of boxed area in A and B. Yellow arrows point to elongated mitochondria in uninfected cell. (E-F) Soft X-ray tomogram slices taken from infected cells depicting viruses at cell edge (E) (black arrows) and abundant DMVs (F). (G) A montage of four tomograms depicting cytoplasmic invasion in an infected cell. Nuclear envelope is outlined in cyan. Nuc, nucleus; Cyto, cytoplasm. Black dots in A and C are gold fiducial markers. Scale bar is 5 µm in A, B, C, D; 1 µm in E, F and G. Representative of 5 cryoET-confirmed SARS-CoV-2 infected cells and 12 control cells imaged by soft X-ray cryo-tomography.

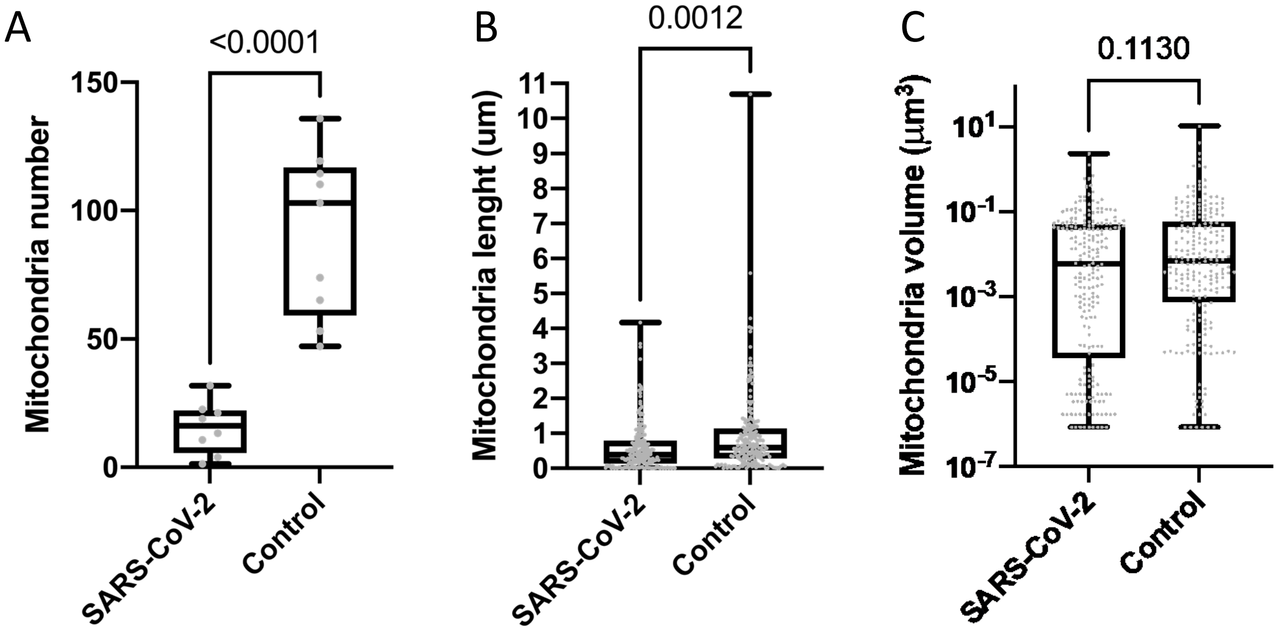

Supplementary Figure 4 | Effect of SARS-CoV-2 infection on mitochondria. (A) Normalized number of mitochondria per tomogram by soft X-ray tomogram (n=8 for SARS-CoV-2 infected and n=9 for control cells), values normalized by cytoplasm area. Box plots represent median, 25% and 75% quartiles, whiskers represent minima and maxima. Individual cell measurements are plotted in gray dots. *p*=0.0006. (B) Mitochondria end-point length in manually segmented serial cryoFIB/SEM volumes (n=200 for SARS-CoV-2 infected and n=208 for control cells). Same type of box plots as in (A). (C) Mitochondria volume in manually segmented serial cryoFIB/SEM volumes (n=221 for SARS-CoV-2 infected and n=228 for control). Same type of box plots as in (A). Statistical test: Two-tailed unpaired Welch's *t* test, *p* values are plotted directly in graphs.

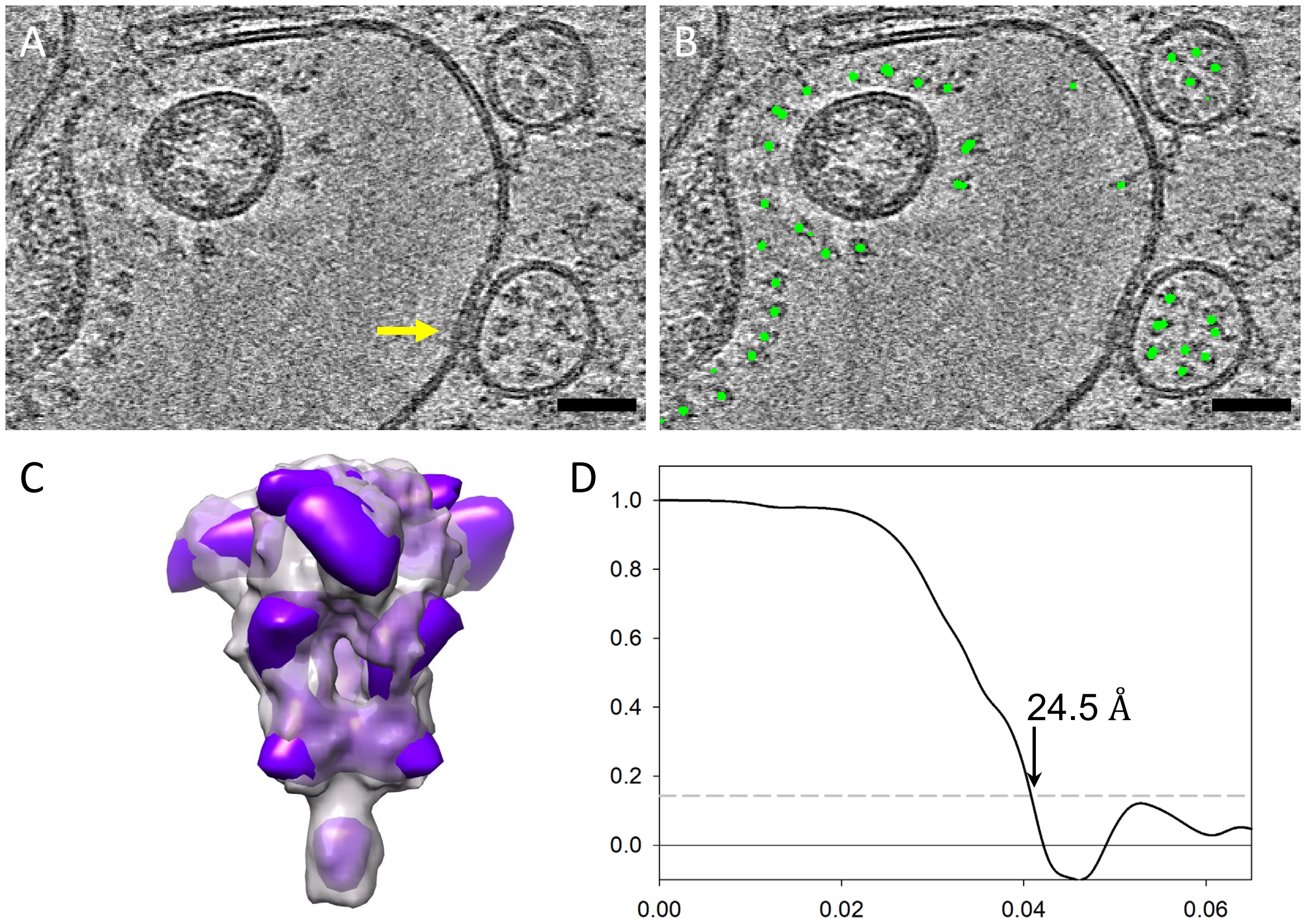

Supplementary Figure 5 | Spike transporting vesicles. (A) A slice of tomogram from a cell lamella, depicting transporting vesicles next to an SMV. There appear electron densities connecting a transporting vesicle to the SMV (yellow arrow). (B) Template matching of prefusion spike (green dots) overlaid with the tomogram slice in A. Spikes were found on the surface of virions, on the membrane of SMVs, and on the transporting vesicles. (C) Subtomogram average of spikes from transporting vesicles (purple) overlapped with the density map of spike from intracellular virus particles (transparent gray). A 600Å-50Å bandpass filter was used during template picking. A total of 42 subtomograms were aligned and averaged. (D) Fourier shell correlation (FSC) plot of subtomogram averaged spike density maps. Scale bar 40 nm.

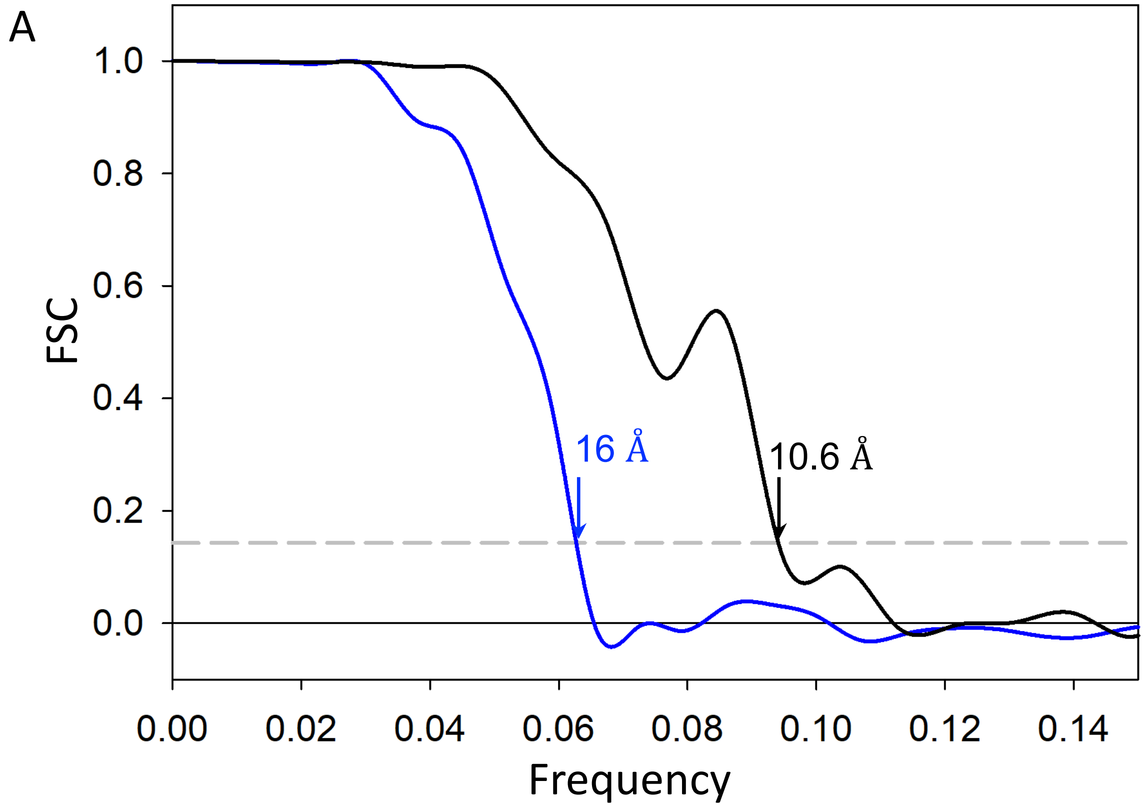

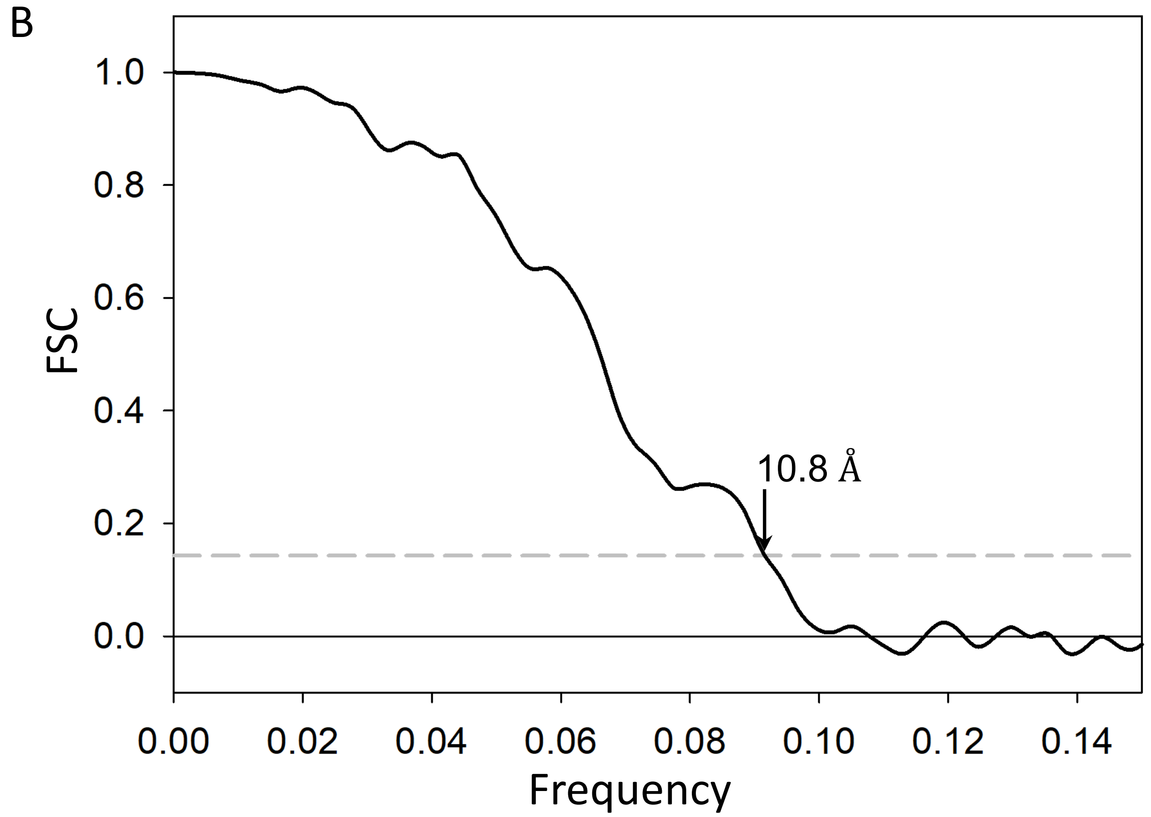

Supplementary Figure 6 | Fourier shell correlation (FSC) plots. (A) FSC between half maps of subtomogram averaged spike density derived from intracellular virions (blue, from 856 subvolumes) and FSC between half maps of extracellular released virions (black, from 2179 subvolumes). (B) FSC between subtomogram averaged spike map derived from extracellular released virions and the published spike map derived from purified virions (EMD-11222) [34]. The dashed lines mark the FSC value of 0.143.

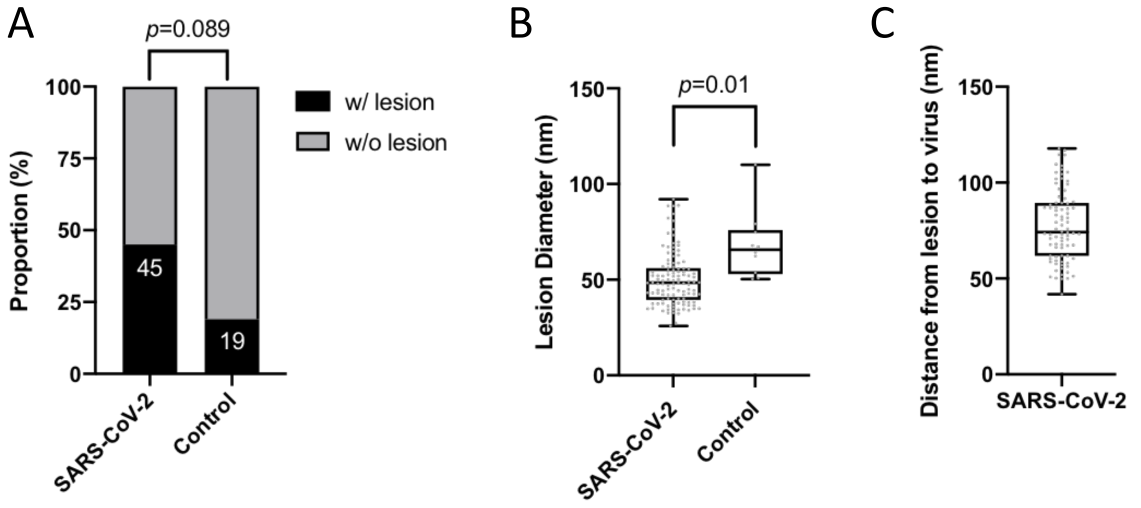

Supplementary Figure 7 | Membrane lesion comparison between SARS-CoV-2-infected and uninfected cells. (A) Percentage of periphery tomograms displaying membrane lesions (33 tomograms from a total of 74 for SARS-CoV-2 infected cells and 3 out of 16 for control). Statistical test: Two-tailed Fisher's exact test. (B) Diameter of lesions in nanometers (n=116 for SARS-CoV-2 infected cells and n=10 for control). Statistical test: Two-tailed unpaired Welch's *t* test. (C) Distance from lesion to closest virus particle (n=84 break/virus pairs). Box plots represent median, 25% and 75% quartiles, whiskers represent minima and maxima. Individual measurements are plotted in grey dots.
